# Modeling the Impact of Agricultural Mitigation Measures on the Spread of Sharka Disease in Sweet Cherry Orchards

**DOI:** 10.3390/plants12193442

**Published:** 2023-09-29

**Authors:** Juan Pablo Gutiérrez-Jara, Katia Vogt-Geisse, Margarita C. G. Correa, Karina Vilches-Ponce, Laura M. Pérez, Gerardo Chowell

**Affiliations:** 1 Centro de Investigación de Estudios Avanzados del Maule (CIEAM), Vicerrectoría de Investigación y Postgrado, Universidad Católica del Maule, Talca 3480112, Chile; mcorreag@ucm.cl; 2 Facultad de Ingeniería y Ciencias, Unidad Adolfo Ibáñez, Diagonal Las Torres 2640, Peñalolén, Santiago 7941169, Chile; 3 Facultad de Ciencias Básicas, Universidad Católica del Maule, Avenida San Miguel 3605, Talca 3480112, Chile; kvilches@ucm.cl; 4 Departamento de Física, Universidad de Tarapacá, Casilla 7D, Arica 1000000, Chile; lperez@academicos.uta.cl; 5 School of Public Health, Georgia State University, Atlanta, GA 30303, USA; gchowell@gsu.edu

**Keywords:** plum pox virus, aphids, mathematical modeling, agricultural management

## Abstract

Sharka is a disease affecting stone fruit trees. It is caused by the Plum pox virus (PPV), with *Myzus persicae* being one of the most efficient aphid species in transmitting it within and among *Prunus* orchards. Other agricultural management strategies are also responsible for the spread of disease among trees, such as grafting and pruning. We present a mathematical model of impulsive differential equations to represent the dynamics of Sharka disease in the tree and vector population. We consider three transmission routes: grafting, pruning, and through aphid vectors. Grafting, pruning, and vector control occur as pulses at specific instants. Within the model, human risk perception towards disease influences these agricultural management strategies. Model results show that grafting with infected biological material has a significant impact on the spread of the disease. In addition, detecting infectious symptomatic and asymptomatic trees in the short term is critical to reduce disease spread. Furthermore, vector control to prevent aphid movement between trees is crucial for disease mitigation, as well as implementing awareness campaigns for Sharka disease in agricultural communities that provide a long-term impact on responsible pruning, grafting, and vector control.

## 1. Introduction

Sharka is one of the most severe diseases that affects stone fruit trees worldwide, caused by the Plum pox virus (PPV) of the genus *Potyvirus* from the family *Potyviridae*. Sharka symptoms range from slight leaf discoloration to tree death [1]. Although sensitivity to PPV can vary among Prunus cultivars, most of them show Sharka symptoms and a certain decrease in productivity [2]. The symptoms shown by infected plants can vary according to the virus strain, the spatial location, the *Prunus* species, the plant tissue, and the season, with spring being the best season to observe symptoms due to temperatures below 30 degrees Celsius [3,4]. Symptoms include other abnormal shapes, colors, or forms of leaves and fruits, premature drop of fruits, fruit lesions, etc. Asymptomatic infection may be more prevalent in warmer climates [5]. In general, infected plants can take several months to show symptoms and their appearance may be temporary and fade during the summer months [3,6].

Disease spread depends on biotic and abiotic vectors. Aphids are the leading biotic vectors of PPV from infested trees to healthy ones present in the orchard or surrounding [3]. More than 20 species of aphids can transmit the virus in a non-persistent manner, of which the species *Myzus persicae* is one of the most efficient in doing so [4]. The intensity of spread depends on several factors, including the efficacy of the aphid species in transmitting and its abundance of winged forms, the winds in the area, and the presence of susceptible cultivars around [5]. The virus can also be transmitted through vegetative propagation techniques in nursery trading such as grafting [3,4,5,7].

Unfortunately, infected trees cannot recover and even if the virus does not kill the plant, they remain PPV-positive. Previous research has reported that virus titers in PPV-positive trees can drop below the PPV detection levels, using ELISA, during warm weather [8]. Hence, infected trees serve as a reservoir for the virus if not eradicated, as well as nearby infected weeds and cultivars, due to the action of virus vectors. Consequently, PPV control relies on public and private prevention measures, such as laws and norms that regulate the trading of vegetative material susceptible to PPV and control illegal exchange trafficking; strict quarantine regulations for nursery stock; chemical vector control; proper eradication of infected material; and the control of weeds and susceptible vegetation [4,5].

Sharka disease was detected in Chile in 1992 and is now distributed in the main Prunus productive regions: Valparaíso, Metropolitana and O’Higgins; it was also recently detected in the Maule region [4]. Chile is the leading sweet cherry exporter worldwide and sweet cherries represent one of the fruits with more economic importance in the country; indeed, the 2022–2023 export season was expected to report nearly 3 billion USD to the country [9].

Mathematical modeling of disease transmission in plants has been developed to improve the understanding, forecasting, and warning, on different scales, of how infections spread in plants and plantations due to viruses, bacteria, and fungi [10]. In particular, epidemiological models of differential equations of the type SIR (susceptible-infectious-removed) and SEIR (susceptible-exposed-infectious-removed) were introduced to model the dynamics of vector-transmitted diseases for different types of plants; for example, citrus [11], banana [12], rice [13], and cereals [14]. Additionally, control techniques are analyzed in such SIR and SEIR models to evaluate the effects of roguing (removal–destruction of diseased plants) and replanting (healthy plants). The main results provide feasible control mechanisms for disease eradication, which could be achieved by implementing realistic roguing and replanting intensities [15,16,17].

In order to include variability in the transmission rates, SIR and SEIR models have included population dynamics and epidemiological aspects of vectors [18]. The interaction of two or more viruses and their coexistence in a host plant system has been modeled [14,18]. Seasonality has been represented by assuming periodic immigration and death rates, and biological control has been modeled by introducing an ecological predator into vector dynamics [19]. Stochasticity and delayed differential equations have been used in models to include uncertainty and time lags between vectors and disease dynamics [20].

The importance of using mathematical modeling to manage Sharka disease has already been highlighted [21]. In this work, we incorporate the impact of agricultural mitigation measures on the spread of Sharka disease in sweet cherry orchards and suggest strategies to prevent agricultural losses.

The proposed model explicitly incorporates risk perception of the farming community towards plant viruses that impacts the effective implementation of agricultural management strategies, which is more likely if individuals in the agricultural community are aware of the existence of a disease and their role in controlling it [22]. Campaigns that can help increase this awareness are a combination of agricultural extension programs that transfer consistent messages, communicate through farmers’ social referents and mass media, and implement group learning and individual communication with farm advisors, as well as new forms of knowledge exchange such as crowd-sourcing, since individuals may not even be aware of plant viruses [22,23]. In general, exposure to agricultural extension services that create awareness and the level of farmers’ education may be crucial for knowledgeable pest management [24].

The following section presents the mathematical model that expresses the dynamics of the Sharka virus, including variables of interest for the study of mitigation strategies. Subsequently, in Section 3, the results of the model are shown. Finally, in Section 4, the discussion and respective conclusions are opened.

## 2. Materials and Methods

Systems of differential equations have been a helpful tool for understanding the evolution of different phenomena. There are dynamics where the evolving process is subject to abrupt changes, represented in pulses. These perturbations have been called impulsive differential equations (IDE) and are used to model ecological, epidemiological, and other problems [25,26,27].

We present a mathematical model of impulsive differential equations to represent the dynamics of Sharka disease, including tree, vector, and human behavior. We denote the total tree population by $N_{T}$ and divide it according to its epidemiological states: susceptible ($S_{T}$), exposed ($E_{T}$), symptomatic infectious ($I_{T}$), asymptomatic infectious ($A_{T}$), and removed ($R_{T}$). This last compartment refers to the trees that have been uprooted. Furthermore, we have divided the vector population ($N_{v}$) into two states: susceptible ($S_{v}$) and infectious ($I_{v}$). Table 1 summarizes the definition of model variables.

**Figure 1:**
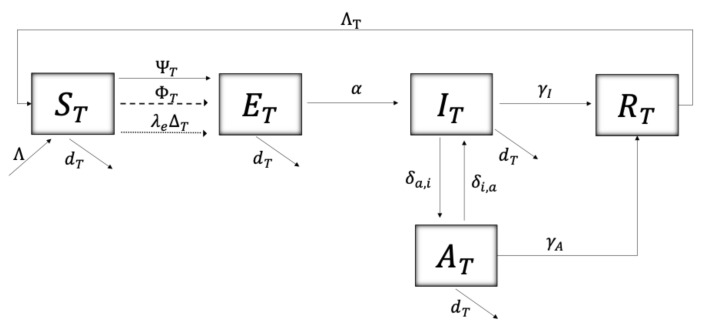
Schematics of the disease dynamics for trees. Table 1 and Table 2 describe, respectively, the variables and parameters used.

The flow diagram in Figure 1 shows the dynamics of the disease in the tree population, where the segmented and dotted lines represent the instants (pulses) in which the continuous dynamics of the disease are altered. A susceptible tree ($S_{T}$) can be infected in three ways: (i) through interaction with an infectious vector ($I_{v}$) at a force of transmission

(1) $$\Psi_{T}=\frac{\beta_{T}^{v,q}}{r^{2}}S_{T}\frac{I_{v}}{N_{T}},$$

where $\beta_{T}^{v,q}/r^{2}$ represents the transmission rate, whose form can be interpreted as a dependence on the movement of the vector ($1/r^{2}$) and a seasonal factor (q=0,1,2,3; for autumn, winter, spring, summer, respectively); (ii) through infected pruning equipment at certain instants (pulses, segmented line) at a force of infection

(2) $$\Phi_{T}=\beta_{T}^{p,q}\frac{P^{*}}{P}S_{T}\frac{A_{T}+I_{T}}{N_{T}},$$

where the transmission rate $\beta_{T}^{p,q}P^{*}/P$, depends on a seasonal factor (*q*) and a dynamic variable P(t), representing the agricultural worker’s risk perception of Sharka disease governed by the differential equation adopted from [28,29,30]

(3) $$P\prime =-\lambda_{1}\left(P-P^{*}\right)+\lambda_{2}\left(I_{T}+R_{T}\right)/N_{T}$$

where $\lambda_{1}$ is the rate of resistance to change, $P^{*}$ is the natural risk perception of the agricultural population and $\lambda_{2}$ is the speed of people’s reactions to the presence of symptomatic trees ($I_{T}$) and removed trees ($R_{T}$); (iii) through the introduction of infected grafts at certain instants (pulses, dotted line) at a rate $\lambda_{e}\Delta_{T},$ where $\Delta_{T}$ is the rate of introduction of grafts and $\lambda_{e}$ is the proportion of infected grafts. The average time that a tree remains exposed is 1/α. The transition rate from an infected tree with symptoms to becoming asymptomatic is $\delta_{a,i}$ and the reverse process occurs at a rate $\delta_{i,a}$. The average time it takes for a symptomatic and asymptomatic tree to be uprooted is given by $1/\gamma_{I}$ and $1/\gamma_{A}$, respectively. $1/\Lambda_{T}$ is the average time it takes for a tree to be replaced by a susceptible (healthy) tree after being uprooted due to Sharka disease. Finally, the natural mortality rate of the tree is given by $d_{T}$, and the recruitment (plantation) by Λ.

**Figure 2:**
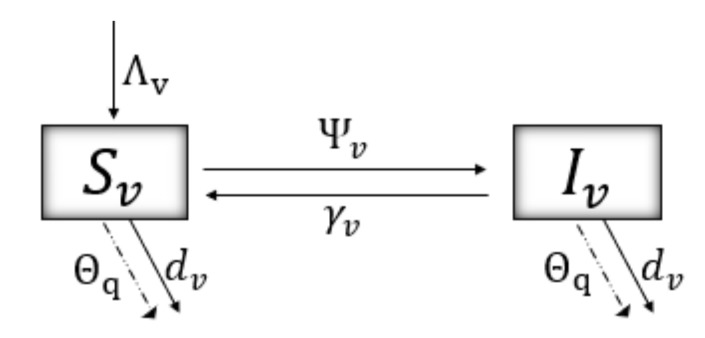
Schematics of the disease dynamics for vectors. Table 1 and Table 2 describe, respectively, the variables and parameters used.

Figure 2 shows the dynamics of the model for the disease in the vector population. The force of infection of a vector when contacting an infectious tree is given by

(4) $$\Psi_{v}=\frac{\beta_{v}^{q}}{r^{2}}S_{v}\left(A_{T}+I_{T}\right)/N_{T},$$

where $\beta_{v}^{q}/r^{2}$ represents the rate of transmission, whose form can be interpreted as a dependence on the movement of the vector ($1/r^{2}$) and a seasonal factor (q=0,1,2,3; for autumn, winter, spring, summer, respectively). An infected vector recovers at a rate $\gamma_{v}$; thus, $1/\gamma_{v}$ is the average time it takes for a vector to remain in its infectious state. $\Lambda_{v}$ and $d_{v}$ correspond to the recruitment and natural mortality rates, respectively. Finally, $\Theta_{q}$ is an additional vector mortality rate, which occurs in certain instants (pulses) due to the implementation of preventive and vector control measures.

The model of impulsive differential equations given by system (5) follows the dynamics from Figure 1 and Figure 2. $t=t_{i}$ and $t=t_{j}$ correspond to the instants in which grafts are introduced and stone tree pruning is performed, respectively. $t=t_{k}$ are the moments of vector control, either by natural or chemical means. $t=t_{n}$ are the instants where awareness campaigns impact risk perception (*P*, see Equation (3)) of the agricultural community towards Sharka disease.

(5) $$\left\{\begin{array}{c} \begin{array}{c} \dot{S_{T}}\left(t\right)=\Lambda -\left(\beta_{T}^{v,q}/r^{2}\right)S_{T}I_{v}/N_{T}+\Lambda_{T}R_{T}-d_{T}S_{T}\\ \dot{E_{T}}\left(t\right)=\left(\beta_{T}^{v,q}/r^{2}\right)S_{T}I_{v}/N_{T}-\left(\alpha +d_{T}\right)E_{T}\\ \dot{I_{T}}\left(t\right)=\alpha E_{T}+\delta_{i,a}A_{T}-\left(\delta_{a,i}+\gamma_{I}+d_{T}\right)I_{T}\\ \dot{A_{T}}\left(t\right)=\delta_{a,i}I_{T}-\left(\delta_{i,a}+\gamma_{A}+d_{T}\right)A_{T}\\ \dot{R_{T}}\left(t\right)=\gamma_{I}I_{T}+\gamma_{A}A_{T}-\Lambda_{T}R_{T}\\ \dot{S_{v}}\left(t\right)=\Lambda_{v}^{q}-\left(\beta_{v}^{q}/r^{2}\right)S_{v}\left(A_{T}+I_{T}\right)/N_{T}+\gamma_{v}I_{v}-d_{v}S_{v}\\ \dot{I_{v}}\left(t\right)=\left(\beta_{v}^{q}/r^{2}\right)S_{v}\left(A_{T}+I_{T}\right)/N_{T}-\left(\gamma_{v}+d_{v}\right)I_{v}\\ \dot{P}\left(t\right)=-\lambda_{1}\left(P-P^{*}\right)+\lambda_{2}\left(I_{T}+R_{T}\right)/N_{T} \end{array}\}\;t\notin \left\{t_{i},t_{j},t_{k},t_{n}\right\}\\ \begin{array}{c} S_{T}\left(t^{+}\right)=S_{T}-\lambda_{e}\Delta_{T}\left(P^{*}/P\right)S_{T}\\ E_{T}\left(t^{+}\right)=E_{T}+\lambda_{e}\Delta_{T}\left(P^{*}/P\right)S_{T} \end{array}\}t=t_{i},\\ \begin{array}{c} S_{T}\left(t^{+}\right)=S_{T}-\beta_{T}^{p,q}\left(P^{*}/P\right)S_{T}\left(A_{T}+I_{T}\right)/N_{T}\\ E_{T}\left(t^{+}\right)=E_{T}+\beta_{T}^{p,q}\left(P^{*}/P\right)S_{T}\left(A_{T}+I_{T}\right)/N_{T} \end{array}\}t=t_{j}\\ \begin{array}{c} d_{v}\left(t^{+}\right)=\left[1+\Theta_{q}\right]\left(P/P^{*}\right)d_{v} \end{array}\}t=t_{k}.\\ \begin{array}{c} P(t^{+})=[1+\mu ]P \end{array}\}t=t_{n}. \end{array}\right.$$

## 3. Results

**Figure 3:**
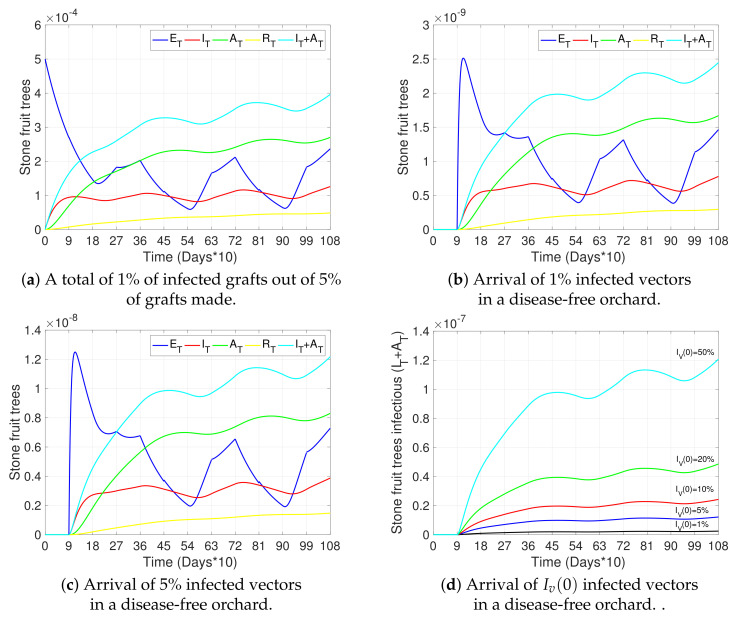
Effects of grafting with infected material and arrival of infectious vectors on disease progress. (**a**–**c**) show the curves representing exposed trees ($E_{T}$, blue), symptomatic infectious trees ($I_{T}$, red), asymptomatic infectious trees ($A_{T}$, green), removed trees ($R_{T}$, yellow), and the total number of infectious trees ($I_{T}+A_{T}$, light blue), for four scenarios: (**a**) infection is only produced by inserting 1% of infected grafts out of 5% of grafts made initially ($t_{i}=0$); (**b**,**c**) infection is only produced by the initial arrival of a percentage of infected vectors ($I_{v}\left(0\right)$) and no grafting with infected material occurs ($\Delta_{T}=0$). Initial conditions were taken: in (**a**) $S_{T}\left(0\right)=1$, $E_{T}\left(0\right)=I_{T}\left(0\right)=A_{T}\left(0\right)=R_{T}\left(0\right)=0$; $S_{v}\left(0\right)=1$, $I_{v}\left(0\right)=0$, $P\left(0\right)=P^{*}$; in (**b**) $S_{v}\left(0\right)=0.99$, $I_{v}\left(0\right)=0.01$ and the remaining as in (**a**); in (**c**) $S_{v}\left(0\right)=0.95$, $I_{v}\left(0\right)=0.05$ and the remaining as in (**a**). (**d**) shows the total number of infectious trees ($I_{T}+A_{T}$) for different initial numbers of infectious vectors ($I_{v}\left(0\right)$), under the same conditions as in (**b**,**c**). All other parameters used in the simulations are as in Table 2.

**Figure 4:**
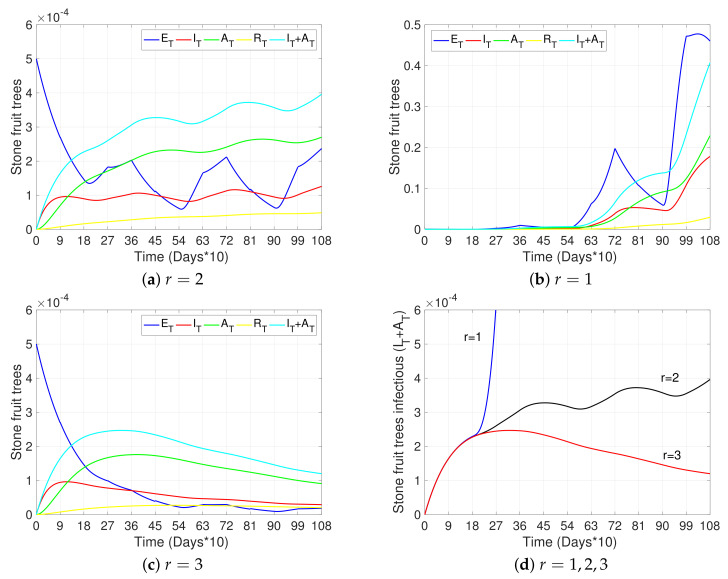
Effects of vector mobility on disease progress. (**a**–**c**) show the curves representing exposed trees ($E_{T}$, blue), infectious symptomatic trees ($I_{T}$, red), infectious asymptomatic trees ($A_{T}$, green), removed trees ($R_{T}$, yellow), and the total number of infectious trees ($I_{T}+A_{T}$, light blue) with respect to time, for r=1, r=2, and r=3, respectively. (**d**) shows the total number of infectious trees ($I_{T}+A_{T}$) with respect to time and for r=1 (blue), r=2 (black), and r=3 (red). The initial conditions were taken as in Figure 3a and all other parameters not specified here and used in the simulations are as in Table 2.

**Figure 5:**
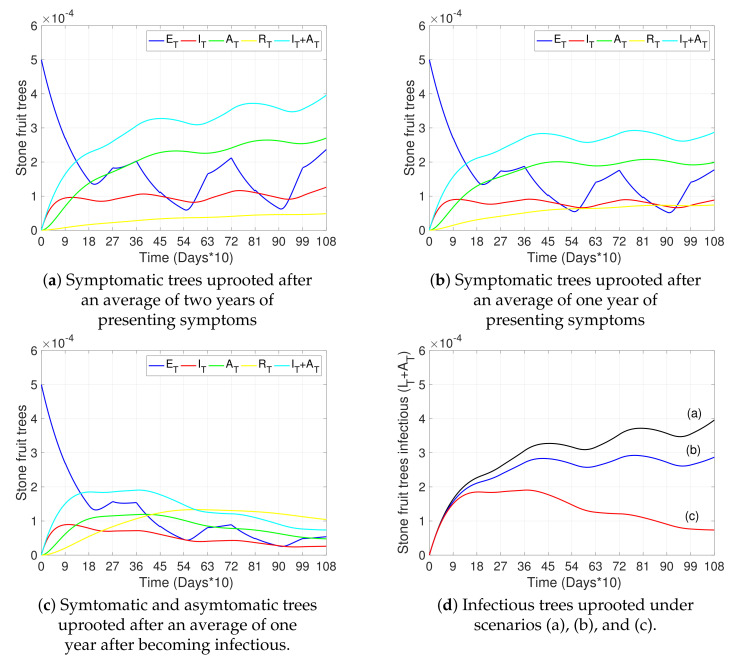
Effects of detection and removal of infected trees on disease progress. (**a**–**c**) show the curves representing exposed trees ($E_{T}$, blue), infectious symptomatic trees ($I_{T}$, red), infectious asymptomatic trees ($A_{T}$, green), removed trees ($R_{T}$, yellow), and the total number of infectious trees ($I_{T}+A_{T}$, light blue) with respect to time. (**a**,**b**) represent scenarios where only symptomatic trees ($I_{T}$) were removed after two years and one year on average, respectively, after presenting symptoms ($1/\gamma_{T}=720$ and $1/\gamma_{T}=360$, respectively). (**c**) represents a scenario in which symptomatic and asymptomatic trees were removed after an average of one year after becoming infectious ($1/\gamma_{T}=360$ and $1/\gamma_{A}=360$, respectively). (**d**) shows scenarios (**a**–**c**) for the total number of infectious trees ($I_{T}+A_{T}$). The initial conditions were taken as in Figure 3a and all other parameters not specified here and used in the simulations are as in Table 2.

**Figure 6:**
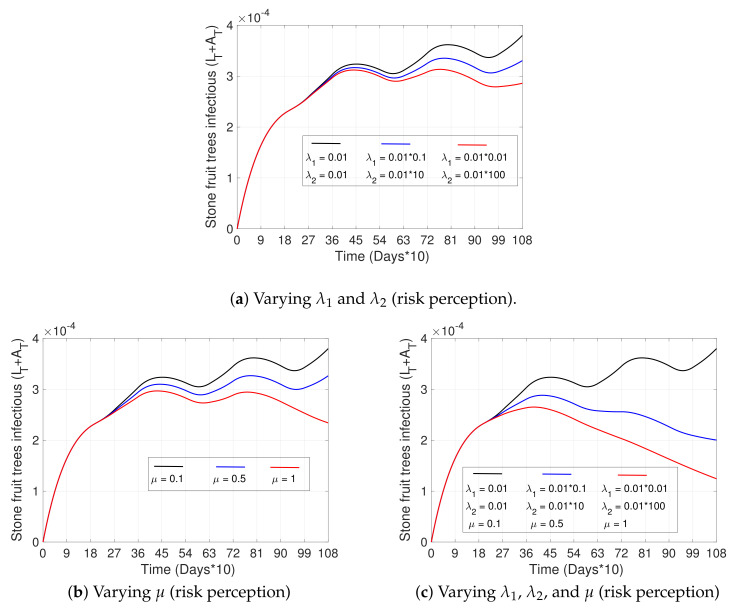
Effects of risk perception on disease progress. (**a**–**c**) show the curve of the total number of infectious trees ($I_{T}+A_{T}$) with respect to time. (**a**) pictures three scenarios for different values of the resistance to change parameter, $\lambda_{1}$, and the rate of reaction to disease, $\lambda_{2}$, from Equation (3), where the black curve represents the smallest level of risk perception, and the red curve the largest. (**b**) shows three scenarios for a varying factor of increase in the risk perception, μ, at instants $t_{n}$ (see Equation (5)), such that the black curve represents the lowest and the red curve the highest effectiveness of awareness campaigns. (**c**) shows scenarios (**a**,**b**) combined. The initial conditions were taken as in Figure 3a, and all other parameters not specified here and used in the simulations are in Table 2.

In this section, we present simulations to show the impact of different risk factors and mitigation measures on the transmission of Sharka disease in cherry trees. Figure 3, Figure 4, Figure 5 and Figure 6 show the dynamics of different states of the trees with respect to time.

Figure 3, Figure 4 and Figure 5a and the black curves in Figure 6 each show a base case scenario, with parameters as in Table 2. In particular, we assume in this scenario that infection is produced only by initial grafting, when the orchard is established ($t_{i}=0$), such that 1% of the total of 5% grafts made per unit of time were infected. Also, this base scenario considers pruning at the beginning of winter and summer ($t_{j}=90$ and $t_{j}=270$ each year, respectively), with a higher transmission rate through pruning in winter than in summer; vector control measures applied at the beginning of spring and summer ($t_{k}=180$ and $t_{k}=270$ each year, respectively), with a greater impact on vector mortality in spring than in summer; and awareness campaigns at the beginning of winter ($t_{n}=90$ each year). Finally, vector–tree transmission rates with r=2 are considered (see Equations (1) and (4)), as well as the fact that infected symptomatic trees were uprooted on average after two years of presenting symptoms ($\gamma_{I}=1/720$) and asymptomatic trees were not detected ($\gamma_{A}=0$). All other sub-figures are compared to this base case scenario.

Figure 3b,c study the effect of vector arrival in a disease-free orchard, where 1% and 5% of vectors arrive infected, respectively. In particular, Figure 3d compares the dynamics of the total number of infectious stone fruit trees ($I_{T}+A_{T}$) over time, for an initial arrival of 1%, 5%, 10%, 20%, and 50%, showing, as expected, a clear increase in total tree infection with an increase in the prevalence of initial disease in vectors. In any case, the impact of the arrival of infected vectors is less than the infection produced by 1% infected grafts (light blue curve in Figure 3a).

Figure 4b,c show the dynamics of the tree states for a scenario where the vector–tree transmission rates are not reduced (vector movement is not considered, r=1), and a scenario where it is reduced by a factor of 1/9 (vector movement becomes more local and limits widespread transmission, r=3), respectively. Figure 4d summarizes the dynamics of the total number of infectious trees ($I_{T}+A_{T}$) in each case: r=1 (widespread transmission), r=2 (base case transmission), r=3 (local spread transmission). As expected, we observe a reduction in the prevalence of the disease in trees with less vector movements. This emphasizes the importance of preventing the development of winged-form aphids by early detection and control of overpopulated trees.

Figure 5 discusses the dynamics of tree states for different removal strategies for infectious trees. Figure 5a,b show the dynamics when only infected symptomatic trees are detected and removed after two years and one year on average, respectively. Figure 5c shows the dynamics when symptomatic and asymptomatic trees are detected and removed on average within one year after the onset of the disease. Figure 5d illustrates, in one plot, the curves of the total number of infectious trees ($I_{T}+A_{T}$) for the three scenarios described previously (from Figure 5a–c). We can observe a significant approximate reduction of 80% by the third year in the total number of infectious trees compared to the base case (red vs black curve) when detecting and uprooting not only symptomatic trees, but also asymptomatic trees early after the onset of the disease.

Figure 6 shows the impact that improving risk perception can have on the disease. We observe that after increasing the speed of reaction ($\lambda_{2}$) and decreasing the resistance to change ($\lambda_{1}$)—parameters that appear in Equation (3) of the risk perception variable P(t)—the percentage of infected trees decreases by approximately 25% (red curve) by the third year (Figure 6a), compared to the base case (black curve). Regarding the effectiveness of prevention campaigns (increasing μ), which are applied at instants $t_{n}$, and keeping $\lambda_{1}$ and $\lambda_{2}$ fixed, we can observe from Figure 6b that the percentage of infected trees by the third year decreases by approximately 35% (black vs red curve). Finally, in the case that campaigns are effective (increasing μ at certain instants) and resistance to change improves along with the speed of reaction in people (decreasing $\lambda_{1}$ and increasing $\lambda_{2}$, respectively, remaining in time), the decrease in infected trees is significant, reaching a reduction in infected trees of more than 50% by the third year (see Figure 6c, black vs red curve).

## 4. Discussion and Conclusions

We have proposed a mathematical model of impulsive differential equations to express the dynamics of the disease among trees and vectors. We obtained results that can serve as input for decision making on mitigation measures against the spread of the virus. Grafting, pruning, and vector control occur as pulses at certain instants and depend on a dynamic human behavior toward the presence of the disease. Human behavior is, in particular, affected by pulse disease awareness campaigns [22,23,24]. In addition, we consider the movement and seasonality of vectors that affect disease transmission. Although several aphid species have been identified as Sharka’s vectors [44], here we used *Myzus persicae* as the main aphid vector, considering that it is the main aphid present in Chilean Prunus orchards [45].

In the proposed model, three routes of Sharka infection are considered between trees: grafting, pruning, and through vectors (aphids). Our results show that grafting with infected material can be critical for the spread of the virus, even more so than the arrival of infectious vectors (see Figure 3a,d). Therefore, increasing people’s risk perception for the disease that reduces grafting with infectious vegetative material could significantly lower disease prevalence (see Figure 6 black curve vs blue and red curve). Hence, agricultural management strategies are suggested to be developed to monitor and test vegetative material for grafting, and awareness campaigns that motivate the agricultural community to purchase grafting material from certified sellers are proposed to be evaluated. Additionally, the arrival of infectious vectors is also a significant problem that increases disease prevalence (see Figure 3d). Although there are orchards with rigorous surveillance and management for PPV vectors, neighboring farms may not provide the same conditions. The latter become a constant reservoir of the disease, which can be easily transferred through vector mobility to Sharka-free orchards.

Climatic conditions, orchard maintenance, and vegetation around the property, among others, predetermine the movement of vectors. Our results describe the impact of vector movement on disease spread (see Figure 4). Thus, to avoid vector migration to other trees, preventing the development of winged-form vectors may be critical in keeping the disease controlled. Since vector overpopulation, as well as other environmental factors, favor the appearance of winged-form aphids and aphid movement [38,39], applying vector control measures is essential for mitigation and prevention measures.

Trees, in general, show symptoms of possible infection through their leaves. However, detecting symptoms by visual inspection with the naked eye alone is complex and certainly misses asymptomatic infectious trees. The results of our model show the need to detect infectious trees (symptomatic and asymptomatic) in the short term; otherwise, the spread of the disease within the orchard may increase significantly (Figure 5), which can produce considerable economic losses. Therefore, growers should have simple and feasible technological monitoring mechanisms to detect the virus in their orchards, especially to detect asymptomatic trees, which are a constant reservoir of the disease. From an agronomic point of view, the use of molecular tools to provide an early diagnostic of the disease in the orchard should be implemented routinely [46,47]. Another diagnostic tool that needs to be studied further is the use of drones to detect symptomatic trees, as these technologies are becoming widely used and have already been useful for other sweet cherry diseases [48].

The risk perception of individuals in agricultural communities towards this disease depends on multiple factors: social, psychological, and economic, among others. Our model considers a change in risk perception according to a dynamic law (see Equation (3)), in which we have tried to encompass these factors into two: the resistance to change while reducing risk perception ($\lambda_{1}$) and the speed of reaction to disease prevalence ($\lambda_{2}$), while increasing risk perception. Resistance to change can be associated with work, health, housing, education, food security, beliefs, etc., subject to the unique circumstances around farmers’ goals and agricultural contexts [22]. The reaction speed can be related to awareness campaigns, such as information via the media, educational interventions, and socialization, among others. In our model, risk perception impacts the efficiency and responsible implementation of vector control, pruning and grafting, and, hence, it impacts the dynamics of the disease (see system (5)). When a population experiences a lower resistance to change and a higher reaction speed, we can observe a decrease in disease prevalence (see Figure 6a). In addition, we have incorporated into our model the effect of awareness campaigns that help to increase risk perception (through $\lambda_{2}$). Our results show the impact of these campaigns on the number of infectious trees, reducing it considerably (see Figure 6b). In particular, when awareness campaigns have a long-lasting effect—reducing in the long-term the resistance to change and increasing the speed of reaction—we observe a reduction in the curve representing infectious trees by more than 50% of the prevalence expected without awareness campaigns in a three-year period (see Figure 6c). A higher risk perception for this disease may encourage growers to implement strategies to prevent its transmission. Therefore, we hope that after awareness campaigns that impact risk perception, aphid control [40], virus reservoirs such as weeds [49] and wild Prunus will be managed [50], and, last but not least, we hope that growers understand the importance of healthy plant material for the establishment of the Prunus orchard [51].

In the model presented through impulsive differential equations, environmental, economic, and dispersion factors, among others, were not involved, but may affect disease transmission dynamics. For example, it would be of interest to include aphid characteristics that can affect propagation, such as that winged aphids may facilitate plant infection [52] or that aphid abundance and feeding may be determined by the presence of natural predators [39]. Therefore, to evaluate the impact of these variables and others on the spread of the virus would enrich future work on the transmission and control of Sharka disease.

## Figures and Tables

**Table 1:** Description of the variables of the model given in system (5).

Variable	Description	Units *
$S_{T}\left(t\right)$	Susceptible trees at time *t*	T
$E_{T}\left(t\right)$	Exposed trees at time *t*	T
$I_{T}\left(t\right)$	Infectious symptomatic trees at time *t*	T
$A_{T}\left(t\right)$	Infectious asymptomatic trees at time *t*	T
$R_{T}\left(t\right)$	Removed (uprooted) trees at time *t*	V
$S_{v}\left(t\right)$	Susceptible vectors at time *t*	V
$I_{v}\left(t\right)$	Infectious vectors at time *t*	V
P(t)	Human risk perception to Sharka disease at time *t*	R

* T:= tree, V:= vector, R:= Risk perception.

**Table 2:** Description of the parameters of the model given in system (5).

Param.	Description	Units *	Baseline	Value	Ref. ^+^
Tree					
$\beta_{T}^{v,q}$	Transmission rate to tree through vector, on season $q^{\;**}$.	$d^{-1}$	$\left[0,\frac{1}{5}\right]$	$\frac{1}{5}*\left[0,\frac{1}{8},1,\frac{1}{2}\right]$	[31]
$\beta_{T}^{p,q}$	Transmission rate to tree through pruning, on season $q^{\;**}$.	$d^{-1}$	$\left[0,\frac{1}{100}\right]$	$\frac{1}{100}\left[0,1,0,\frac{1}{3}\right]$	
1/α	Average virus-PPV incubation time.	*d*		$\frac{31}{0.17}$	[32]
$1/\gamma_{I}$	Average time until a symptomatic infectious tree is uprooted.	*d*		$\frac{1}{720}$	C.A.
$1/\gamma_{A}$	Average time until an asymptomatic infectious tree is uprooted.	*d*		0	C.A.
$\delta_{a,i}$	Transition rate from symptomatic to asymptomatic infectious.	$d^{-1}$	$\left[\frac{1}{90},\frac{1}{60}\right]$	$\frac{1}{70}$	[6]
$\delta_{i,a}$	Transition rate from asymptomatic to symptomatic infectious.	$d^{-1}$		$\frac{1}{240}$	[6]
$\Lambda_{T}$	Replacement rate of removed trees.	$d^{-1}$		$\frac{1}{360}$	[33]
Λ	Recruitment rate.	$T*d^{-1}$	$N_{T}*d_{T},N_{T}=1$	$\frac{1}{7200}$	
$\Delta_{T}$	Grafting rate.	$d^{-1}$		$\frac{5}{100}$	[34,35]
$\lambda_{e}$	Proportion of infected grafts.	Unitless	$\left[0,1\right]$	$\frac{1}{100}$	C.A.
$d_{T}$	Natural mortality rate	$d^{-1}$	$\left[\frac{1}{35},\frac{1}{15}\right]\frac{1}{365}$	$\frac{1}{7200}$	[36]
$t_{i}$	Time instants where grafting occurs.	*d*		0	[34,35]
$t_{j}$	Time instants where pruning occurs.	*d*		90(1+2k), $k\in N_{0}$	[37]
Vector					
$\beta_{v}^{q}$	Transmission rate to vector, on season $q^{\;**}$.	$d^{-1}$	$\left[0,\frac{13}{100}\right]$	$\frac{13}{100}*\left[1,1,1,1\right]$	[31]
$1/r^{2}$	Vector–host transmission rate reduction due to vector movement.	Unitless		$\frac{1}{4}$	[38,39]
$1/\gamma_{v}$	Average time a vector remains infectious.	*d*		0.1	[40]
$\Lambda_{v}$	Recruitment rate.	$V*d^{-1}$	$N_{v}*d_{v},N_{v}=1$	$\frac{1}{30}$	
$d_{v}$	Natural mortality rate.	$d^{-1}$		$\frac{1}{30}$	[41,42]
$\Theta_{q}$	Mortality rate increase factor, on season $q^{\;**}$.	Unitless		$\left[0,0,\frac{3}{10},\frac{3}{100}\right]$	C.A.
$t_{k}$	Time instants where vector control occurs.	*d*		180(2k+1); 90+180(2k+1), $k\in N_{0}$.	[33]
Human					
$\lambda_{1}$	Rate of resistance to change risk perception.	$d^{-1}$	$\left[0,1\right]$	0.01	[29,30,43]
$\lambda_{2}$	Per capita reaction rate to change risk perception.	$R*d^{-1}$	$\left[0,1\right]$	0.01	[29,30,43]
$P^{*}$	Natural risk perception.	*R*		0.5	[29,30,43]
μ	Risk perception increase factor.	Unitless		0.1	C.A.
$t_{n}$	Time instants where educational prevention campaigns occur.	*d*		90(1+4k), $k\in N_{0}$	C.A.

* d:= day, T:= tree, V:= vector. ** *q* = 1(autumn), 2(winter), 3(spring), 4(summer), ^+^ C.A. = Chosen by author.

## Data Availability

Not applicable.
